# Cutaneous Squamous Cell Carcinoma in Patients with Hidradenitis Suppurativa

**DOI:** 10.3390/cancers13051153

**Published:** 2021-03-08

**Authors:** Elysia Racanelli, Abdulhadi Jfri, Amnah Gefri, Elizabeth O’Brien, Ivan V. Litvinov, Andrey Zubarev, Evgeny Savin, Elena Netchiporouk

**Affiliations:** 1 Faculty of Medicine, University of Montreal, Montreal, QC H3T 1J4, Canada; elysia.racanelli@umontreal.ca; 2 Division of Dermatology, McGill University Health Centre, Montreal, QC H3G 1A4, Canada; adulhadi.jfri@mail.mcgill.ca (A.J.); ivan.litvinov@mcgill.ca (I.V.L.); andrey.zubarev@muhc.mcgill.ca (A.Z.); McGill_Ca@outlook.com (E.S.); 3 Department of Dermatology, Al-Noor Specialist Hospital, 24242 Makkah, Saudi Arabia; ahgefri@moh.gov.sa

**Keywords:** hidradenitis suppurativa, Verneuil’s disease, acne inversa, cutaneous squamous cell carcinoma, Marjolin ulcer, human papillomavirus

## Abstract

**Simple Summary:**

Cutaneous squamous cell carcinoma (cSCC) is a recognized but infrequent complication of hidradenitis suppurativa (HS). After performing a thorough literature review of all the published cases of cSCC developing in HS, we identified that White males who are smokers and afflicted with severe gluteal or perianal HS are more likely to develop cSCC. Human papillomavirus (HPV) was found to be a common co-factor. cSCC of ulcerative morphology with a poorly differentiated histologic grade, the presence of nodal or distant metastasis, and recurrent cSCC were associated with decreased survival. Regular screening of suspicious lesions for detection of cSCC in patients with HS, notably those with the aforementioned demographics, is highly recommended.

**Abstract:**

*Background*: Cutaneous squamous cell carcinoma (cSCC) is a rare complication of hidradenitis suppurativa (HS). *Objectives*: To conduct a systematic review and an individual patient data (IPD) meta-analysis to describe the clinical characteristics of HS patients developing cSCC and determine predictors of poor outcome. *Methods*: Medline/PubMed, Embase, and Web of Science were searched for studies reporting cSCC arising in patients with HS from inception to December 2019. A routine descriptive analysis, statistical hypothesis testing, and Kaplan–Meier survival curves/Cox proportional hazards regression models were performed. *Results*: A total of 34 case reports and series including 138 patients were included in the study. The majority of patients were males (81.6%), White (83.3%), and smokers (n = 22/27 reported) with a mean age of 53.5 years. Most patients had gluteal (87.8%), Hurley stage 3 HS (88.6%). The mean time from the diagnosis of HS to the development of cSCC was 24.7 years. Human papillomavirus was identified in 12/38 patients tested. Almost 50% of individuals had nodal metastasis and 31.3% had distant metastases. Half of the patients succumbed to their disease. *Conclusions*: cSCC is a rare but life-threatening complication seen in HS patients, mainly occurring in White males who are smokers with severe, long-standing gluteal HS. Regular clinical examination and biopsy of any suspicious lesions in high-risk patients should be considered. The use of HPV vaccination as a preventive and possibly curative method needs to be explored.

## 1. Introduction

Hidradenitis suppurativa (HS) is a common chronic inflammatory skin disease that leads to the formation of painful nodules, abscesses, and/or fistulas in body folds. It is thought to start from follicular occlusion and dilation of the pilosebaceous unit with rupture of the hair follicle, whose contents trigger an inflammatory response and cause an abscess to form [1,2]. While its pathogenesis remains to be elucidated, it is commonly associated with smoking, metabolic syndrome, acne tetrad, and inflammatory comorbidities such as Crohn’s disease and arthritis [3,4,5]. The clinical severity of HS is most commonly graded according to the Hurley classification, defining stage 1 as non-scarring transient abscesses; stage 2 as recurrent abscesses with sinus tract formation and early scarring; and stage 3 as multiple, coalescent abscesses and interconnected sinus tracts along with extensive scarring [3].

The complications associated with HS are usually seen in severe and long-standing disease (i.e., Hurley stage 3) and can either be due to systemic inflammation (e.g., anemia of chronic disease, amyloidosis) or local tissue destruction including lymphedema, decreased limb range of motion due to scarring, anogenital strictures or fistulas, and cutaneous squamous cell carcinoma (cSCC) [3]. A subset of cSCC may arise within an area of chronic inflammation or a pre-existing scar, known as the Marjolin ulcer (MU). MU is a rare but potentially life-threatening complication of HS due to its increased metastatic potential and high lethality [6]. In a nationwide Swedish registry, 4.6% of HS patients developed MU as a complication of their disease [7]. However, the true prevalence of MU in HS is difficult to ascertain as HS is often underdiagnosed by non-dermatology physicians; thus, many patients may not have received a diagnosis of HS prior to the detection of cSCC [8]. Further, cSCC is rarely reported to cancer registries making this complication seldom known to many healthcare providers. It is critical for physicians to maintain a high index of suspicion and understand the risk factors and clinical features of cSCC in HS patients to allow a timely diagnosis and proper management of this complication. For this reason, we performed an individual patient data (IPD) meta-analysis of case reports and series published to date to better understand the clinical and demographic characteristics of HS patients developing cSCC, prognosis, and predictors of lethality.

## 2. Methods

### 2.1. Study Identification

**Figure 1:**
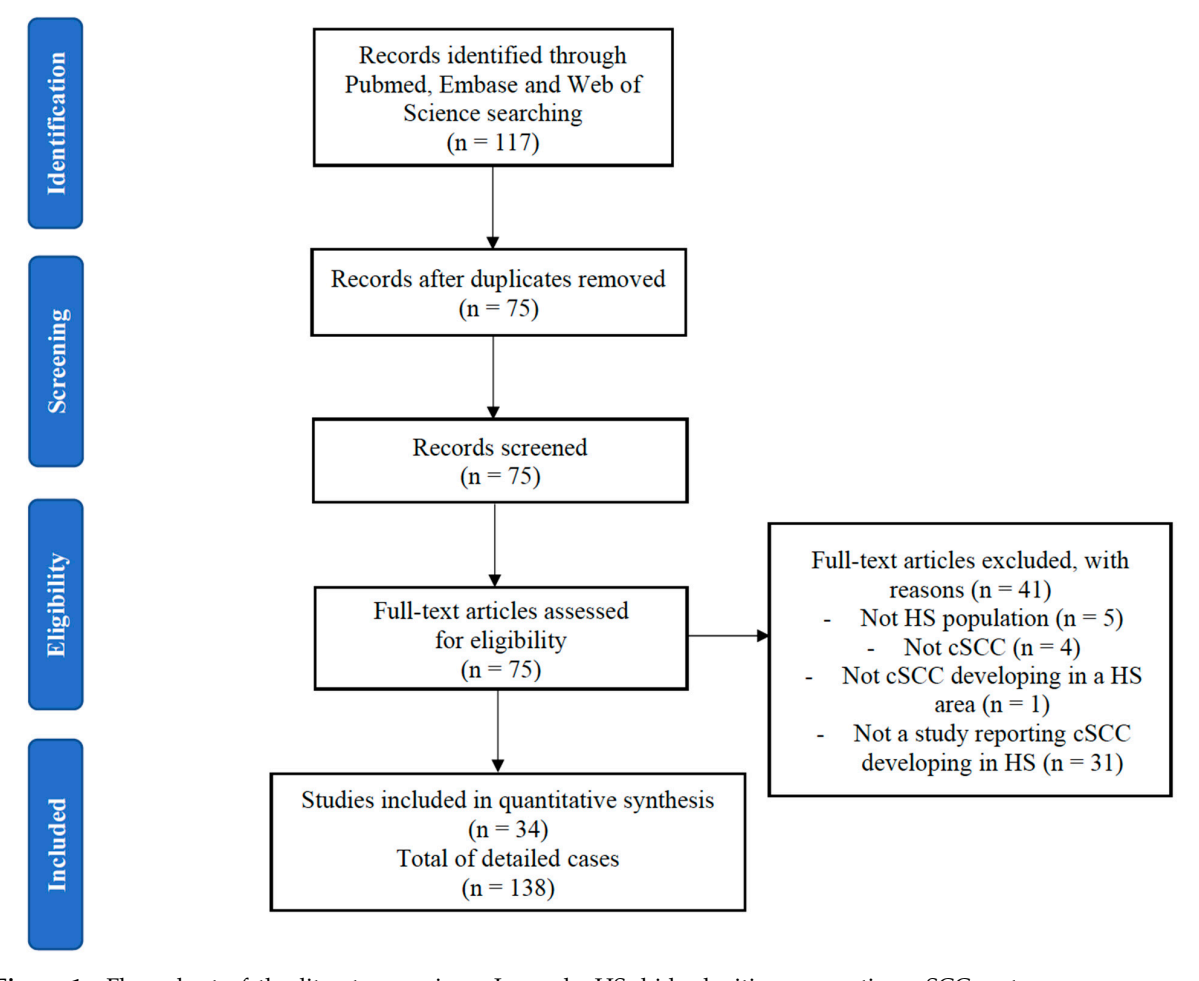
Flow chart of the literature review. Legend. HS: hidradenitis suppurativa; cSCC: cutaneous squamous cell carcinoma.

Medline/PubMed, Embase, and Web of Science were searched in December 2019 for studies reporting cSCC arising in HS patients from inception (without date limitation) using: “squamous cell carcinoma”, “SCC” OR “Marjolin ulcer” AND “hidradenitis suppurativa”, “HS”, “Verneuil’s disease” OR “acne inversa”. The initial search retrieved 117 studies (Figure 1). After manual curation, 75 studies were retained for full text screening for inclusion/exclusion criteria. Articles in which patients were ≥12 years old and had developed cSCC within an HS-ridden area were included. Exclusion criteria were cSCC in a non-HS area, cSCC occurrence before the clinical diagnosis of HS, and conference abstracts/papers which were not published in a peer-reviewed journal, with articles in non-English, French, or Spanish language. A total of 34 papers were included for detailed analysis [2,6,9,10,11,12,13,14,15,16,17,18,19,20,21,22,23,24,25,26,27,28,29,30,31,32,33,34,35,36,37,38,39,40]. No additional studies were identified by reviewing citations of retrieved articles. Two authors (ER and EN) reviewed all the abstracts and manuscripts for pertinence. In case of disagreement, a third author was consulted (AJ).

### 2.2. Quality Assessment

Methodological quality appraisal was performed using a tool proposed by Murad et al. [41] designed for case reports and series consisting of 8 questions to evaluate selection, ascertainment, causality, and reporting (Supplementary Table S1). Three of the 8 questions were excluded as they were irrelevant or not feasible in regard to the study population (HS) and the outcome evaluated (cSCC), as these questions were mostly applicable to adverse drug reactions.

### 2.3. Statistical Analysis

A routine descriptive analysis, statistical hypothesis testing, and Kaplan–Meier survival curves/Cox proportional hazards regression models were performed using SPSS and SAS UE software. Specifically, the following variables were studied for association with risk of death: age at time of diagnosis, sex, race, smoking status, human papillomavirus (HPV) status, HS duration, HS location and severity (Hurley staging), cSCC morphology and histopathological grade, concomitant immunosuppression, lymph node and organ metastasis, type of cSCC treatment, and cSCC recurrence. A *p*-value of <0.05 was considered statistically significant.

## 3. Results

All the case reports and series met the minimum quality assessment standard and were included in the meta-analysis (Supplementary Table S1) [2,6,9,10,11,12,13,14,15,16,17,18,19,20,21,22,23,24,25,26,27,28,29,30,31,32,33,34,35,36,37,38,39,40]. Selection bias cannot be excluded as most authors did not explicitly explain their selection method; therefore, there is a possibility that some cases of cSCC arising from HS wounds were not reported, underestimating the total number of cases currently in the literature. Exposure (HS) and outcome (development of cSCC) were ascertained in all the studies, with most papers displaying pictures of the patients’ lesions, making it verifiable by the reader. Follow-up was deemed acceptable in all the studies as it pertained to history of HS before cSCC diagnosis, which was 24.7 years of duration on average. While a few authors were not very elaborate in their case description, most thoroughly described the patient demographics, the histological details, and the attempted treatments. Due to these reasons, we judged the overall methodological quality of the selected articles to be satisfactory.

### 3.1. Patient Demographics

A total of 138 cases met the inclusion criteria. Patient characteristics are detailed in Table 1. The majority of patients were males (81.6%) and White (83.3%). Surprisingly, only 14 (10%) patients reported the use of immunosuppressive systemic therapy for their HS including prednisone, azathioprine, cyclosporine, and/or biologic therapies (e.g., TNF-α inhibitors). Specifically, 3 patients were previously treated with adalimumab and 4 with infliximab. Only 3 patients received double immunosuppression, where 2 patients were receiving adalimumab with cyclosporine, azathioprine, and/or prednisone and 1 patient being treated with infliximab and prednisone. Concomitant Crohn’s disease was reported in 4 patients (2.9%).

### 3.2. cSCC Characteristics

The most common morphology of cSCC developing in HS was an ulcer (68.8%) followed by a nodule/plaque (15.6%) or a verrucous lesion (15.6%). Histologic grades were well-differentiated (45.9%), verrucous (14.3%), moderately differentiated (25.5%), poorly differentiated (12.2%), and in situ disease (2.0%) (Table 1).

### 3.3. Associations/Risk Factors with Cutaneous Squamous Cell Carcinoma

At the time of cSCC diagnosis, the average age was 53.5 years ± 10.2 and the mean lead time from onset of HS to cSCC was 24.7 years ± 11.9. The vast majority of patients had severe HS (Hurley stage 3, 88.6%), only a few had Hurley stage 2 (10.1%), and 1 patient had Hurley stage 1 (1.3%). The most frequently affected site was gluteal/perianal (87.8%). Rarely, patients presented with vulvar, scrotal, scapular, inguinal, and axillary cSCC. In studies where smoking history was obtained, 81.5% (22/27 patients) were active or past smokers [2,6,18,19,25,28,35,36,37,40].

Ten studies (36 patients) investigated the presence of HPV by either DNA and RNA in situ hybridization, polymerase chain reaction (PCR) or p16 staining on paraffin-embedded tumor specimens [2,6,15,17,18,19,20,25,35,36]; HPV was detected in 10 of these patients. In 2 additional patients, HPV infection was confirmed visually given the presence of condyloma acuminata and vulvar intraepithelial neoplasia [42,43]. High-risk HPV (e.g. HPV-16) was the most prevalent, in a total of 9 tested patients [7,17] (Table 1). There is no data regarding HPV vaccination status in any of the reported patients.

### 3.4. Staging of Squamous Cell Carcinoma and Predictors of Adverse Outcome

**Figure 2:**
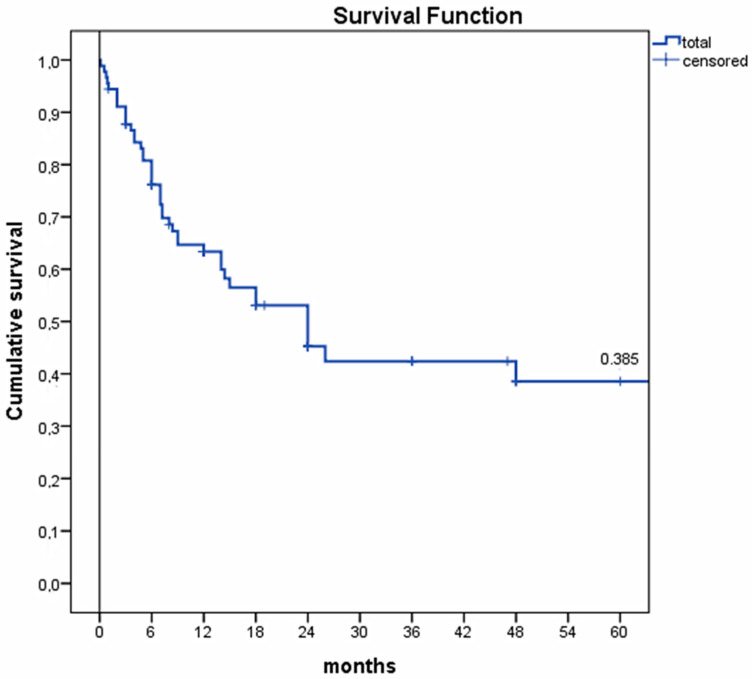
Kaplan–Meier survival curve. Legend. One- and 5-year survival rates were 63.4% and 38.5%, respectively. The factors with the greatest positive association with increased risk of death were male sex (*p* = 0.002), immunosuppressive therapy (*p* < 0.0001), presence of metastasis (*p* = 0.016), and recurrent cSCC at a different site *p* < 0.023.

Nodal metastases, predominantly inguinal, were detected in 43 patients (46.2%). Distant metastases were found in 31 patients (31.3%), mainly in the lungs. Tumor node metastasis (TNM) staging was directly reported for only 12 patients from 5 studies [6,15,22,25,36]. In total, 100/131 patients (76.3%) had undergone surgical excision of the tumor, 37/50 patients (74%) received radiotherapy, and 26/43 (60.5%) received polychemotherapy. cSCC recurred at the excision site in 30/76 patients (39.5%). The lethality rate secondary to cSCC and/or its complications was 50.9% (56 patients); in 4 additional cases, demise was deemed unrelated to cSCC (3.6%) (Table 1). Using Kaplan–Meier curves, 1- and 5-year survival rates were 63.4% and 38.5%, respectively (Figure 2).

To assess the factors influencing survival time and predictors of lethal outcome, the Cox proportional hazard model was used (Table 2). Among factors negatively affecting the likelihood of survival, we observed the presence of ulcerative morphology, which was associated with a 10-fold increased lethality as opposed to verrucous morphology (*p* = 0.023). Not surprisingly, the risk of death correlated with the histologic grade and clinical stage of the disease; poorly differentiated cSCC increased the risk of death by 7.2-fold (*p* < 0.0001), presence of nodal metastasis by 9.7-fold (*p* < 0.0001), and the presence of distal metastasis by 4.3-fold (*p* < 0.0001). Patients with cSCC surgically excised performed better than those needing radiotherapy and/or polychemotherapy (odds ratio [OR], 8.2, *p* < 0.0001). When cSCC recurred post-treatment either at the excision site (OR 5.2, *p* < 0.05) or at a nearby location (OR 7.9, *p* < 0.05), the risk of death significantly increased. Using available data, no statistically significant difference could be demonstrated for HS severity and duration, disease location, smoking, and/or HPV status. Despite a trend for a worse outcome, a definitive conclusion could not be made regarding the use of immunosuppressive therapy for HS (e.g., cyclosporine, azathioprine, TNF-α inhibitors) and risk of death because the total number of patients treated (14) was too small to demonstrate a statistically significant difference. While female sex and White race had a favorable prognostic trend, results were not statistically significant (*p* > 0.05).

## 4. Discussion

cSCC is the second most common skin cancer affecting approximately 1 million individuals yearly in the United States of America [44]. While, the most important risk factors for the development of cSCC are cumulative sun exposure, age, fair skin, and immunosuppression, it has been described to arise in chronic wounds (e.g., trauma, burn), chronic inflammatory processes (e.g., hypertrophic lichen planus) or sclerosing conditions (e.g., systemic sclerosis or morphea [45,46,47,48]. Regardless of the context, cSCC commonly carries a mutation in the tumor suppressor protein 53 (TP53) [49].

While the pathogenesis of cSCC arising in chronic wounds/inflammatory conditions is different from cSCC arising in chronically sun-exposed sites, its precise mechanisms remain unknown. A theory elaborated by Fabbrocini to explain the occurrence of cSCC in HS is the “immunocompromised cutaneous district”. This concept stipulates that a region of chronically diseased skin has a locally dysfunctional immune control, which in turn allows for the development of a tumor or an infection [50]. Stasis of lymphatic drainage, a known complication of HS, is a contributing factor to this phenomenon as it prevents the normal flow of immune cells into the lymphedematous region and thereby increases the risk for malignant transformation [50,51]. Further, the tumor growth within a chronic non-healing wound is potentiated by the growth factor rich stroma (e.g., platelet-derived growth factor (PDGF), vascular endothelial growth factor (VEGF), etc.) [52,53,54].

Of the 138 published cases analyzed in this study, cSCC almost always (˃80%) occurred in White males with Hurley stage 3 and long-standing gluteal/perianal HS, presenting as an ulcerative lesion. Evidence of HPV was demonstrated in ˃30% of tested patients. Thereby, in addition to chronic inflammation, the potential role of HPV and/or other infectious agents as local co-factors may explain why most cSCC arising in HS were localized to the gluteal area [18]. HPV is well known to play an important role in the pathogenesis of mucosal and skin SCC as it prevents apoptosis, allowing continuous viral DNA replication [55]. HPV E6 protein targets p53 towards proteasomal degradation while viral E7 protein binds retinoblastoma protein (Rb) and promotes its ubiquitination, thus resulting in an inhibition of apoptosis and in a loss of a crucial cell-cycle checkpoint, respectively [56]. Flores et al. noted a significant positive correlation of HPV-16 viral load between proximal anatomic sites in the anogenital region of men, suggesting a possible autoinoculation in male HPV HS patients, facilitated by humidity and inadequate hygiene due to chronic pain in the affected area [57].

Along with its recognized safety and efficacy against HPV oncogenic strains, the commercially available HPV vaccines may also offer adjuvant potential in the context of established cancer. Resolution of cancer has been described following vaccination in non-HS-related cSCC [58]. The nonavalent HPV vaccine (^®^Gardasil-9) is approved for the prevention of HPV-related mucosal cancers in women ≤45 and men ≤26 years of age [59], including in genital and perianal areas, which are the regions where cSCC developing in HS most commonly occurs. Our data suggest that HPV may be implicated in at least a subset of cSCC arising in HS wounds; however, evidence is currently lacking regarding the efficacy of HPV vaccination as a preventive and even curative measure in this population, thus additional research is warranted.

Among the studies that reported smoking status in this review, >80% of patients were smokers [2,6,18,19,25,28,35,36,37,40]. An association between smoking and HS is well documented. It was postulated that tobacco use may lead to impaired Notch signaling [18], which is involved in normal hair follicle development and has important immune regulatory functions. When Notch signaling is inhibited, follicular homeostasis is disrupted, leading to hair follicle rupture and a local inflammatory response [2]. Notch also plays a potential tumor suppressive role in cSCC and other keratinocyte carcinomas [60]. Thus, smoking may increase the risk of cSCC in patients with HS via Notch inhibition. While counselling for smoking cessation is already well known to physicians treating HS patients, risk of cSCC may serve as an extra argument.

Over half of the published cases succumbed to their disease, which is similar to the mortality rates described for cSCC developing in other chronic wounds and/or inflammatory conditions [61,62]. In this study, the most important factors related to poor outcome were ulcerative morphology, higher cSCC histologic grade and clinical stage, disease recurrence, and the need for chemotherapy and/or radiation therapy. Additional factors associated with higher mortality that did not reach statistical significance were male sex, African American race, smoking, absence of HPV, older age, and the presence of immunosuppression.

Treatment of cSCC was reviewed elsewhere [6,7]. We wish to highlight that recently, a series of targeted therapies were approved for advanced cSCC including epidermal growth factor receptor (EGFR) inhibitors (e.g., cetuximab) or immunotherapy (e.g., cemiplimab) as neoadjuvant therapy for patients with inoperable or incompletely resected regional disease and for those with regional recurrence or metastatic disease [63,64,65]. This patient cohort did not receive these therapies, likely because they were not available at the time. However, in the future, they can be considered as an important therapeutic approach to improve morbidity and lethality of cSCC developing in HS.

### Limitations

Our study has limitations. Firstly, only case reports and series of cSCC developing in HS have been published and thereby included in our analysis. These studies are prone to publication bias. Reporting of the variables, namely the clinical characteristics and prognostic predictors, was not homogenous among the studies and some variables were missing in the original datasets. On several occasions, we contacted the authors of the original manuscripts, but they no longer had the missing information. Finally, total sample size was 138 patients, which is limited. The need for larger observational studies is undeniable.

## 5. Conclusions

One of the most dreaded complications of severe, long-standing HS is the development of cSCC. So far, 138 cases of cSCC arising in HS have been published, usually occurring in the perianal area of White men who were smokers. cSCC occurring in HS bears an aggressive behavior with its overall 5-year survival of 38.5%. Factors accounting for increased mortality include cSCC of ulcerative morphology, advanced grade and stage, as well as locoregional recurrence. Timely detection is key to improve prognosis; hence, regular total body skin examination with inguinal lymph node palpation should be performed in all severe HS cases. Furthermore, the role of HPV vaccination in the prevention of cSCC, and possibly its treatment, in HS patients is an interesting topic for future research.

## Figures and Tables

**Table 1:** Demographic and clinical characteristics of patients.

Patient Characteristics	Number (n)	%
**Total**	138	100
**Sex (males) (*n* = 125)**	102	81.6
**Age, in years (mean, SD)**	53.5 ± 10.2	
**HS duration prior to cSCC (mean, SD)**	24.7 ± 11.9	
**Race (*n* = 84)**		
White	70	83.3
African-American	12	14.3
Others	2	2.4
**Smoking (*n* = 27)**	22	81.5
**Hurley stage (*n* = 79)**		
1	1	1.3
2	8	10.1
3	70	88.6
**Site of cSCC (*n* = 131)**		
Gluteal and perianal	115	87.8
Inguinal	4	3.1
Vulvar	7	5.3
Scrotal	2	1.5
Scapular	2	1.5
Axillary	1	0.8
**Morphology of cSCC (*n* = 77)**		
Ulcer	53	68.8
Nodule/plaque	12	15.6
Verrucous	12	15.6
**Histological grade of cSCC (*n* = 98)**		
0 (in situ)	2	2.0
1 (well-differentiated)	45	45.9
1(V) (well-differentiated, verrucous)	14	14.3
2 (moderately differentiated)	25	25.5
3 (poorly differentiated)	12	12.2
**Immunosuppressive treatment (*n* = 14)**		
Biologic	4	28.6
Nonbiologic *	6	42.9
Biologic and nonbiologic	3	21.4
None	1	7.7
**Concomitant autoimmune chronic disorder**		
Crohn’s disease	4	2.9
**HPV status (*n* = 38)**		
Lesional HPV detected	12	31.6
High-risk HPV	9	75
**Metastatic status**		
Nodal microscopic metastasis (*n* = 94)	43	46.2
Site = inguinal (*n* = 32)	23	71.9
Distant metastasis (n = 99)	31	31.3
Site = lung (*n* = 28)	11	39.3
**cSCC treatment**		
Surgical excision (*n* = 131)	100	76.3
Radiotherapy (*n* = 50)	37	74.0
Chemotherapy (*n* = 43)	26	60.5
**Recurrence of cSCC post excision (*n* = 76)**		
Same site	30	39.5
Nearby site	2	2.6
**cSCC vital status (*n* = 110)**		
Demise due to cSCC or its complications Demise due to cause unrelated to cSCC Time to demise in months (mean, SD)	56	50.9
	4 11.6 ± 15.2	3.6
**Duration of follow-up in patients with favorable outcome, in months (mean, SD)**	28 ± 31.3	

Legend. SD: standard deviation; n: number of cases reporting the respective variable; cSCC: cutaneous squamous cell carcinoma; HS: hidradenitis suppurativa; HPV: human papillomavirus; high-risk α-HPV types (HPV-16, 18, and 68). * Azathioprine, cyclosporine, or oral prednisone.

**Table 2:** Univariate Cox Proportional Hazards Model.

Variables (Prognostic Factors)	Value	Univariate Cox Proportional Hazards Model		
		OR	95%CI	*p*
Sex (*n* = 89)	2—females	1		
	1—males	1.9	0.673–5.376	0.225
Age (*n* = 87)	Mean—53.6	1		
	>Mean	1.014	0.983–1.046	0.376
Race (*n* = 56)	2—African-American	1		
	1—White	0.612	0.265–1.414	0.251
Hurley score (*n* = 51)	1—1	-		
	2—2	1		
	3—3	2.706	0.336–20.036	0.330
Site of cSCC (*n* = 89)	1—gluteal/perianal/perineal	1		
	2—inguinal	0.000	0.000	0.982
	3—vulvar	0.713	0.097–5.207	0.738
	4—scrotal	0.000	0.000	0.987
	5—scapular	2.970	0.710–12.415	0.136
	6—axillary	-		
Duration of HS prior to cSCC (years) (*n* = 82)	Mean—24.48	1		
	>Mean	1.024	0.998–1.051	0.074
Presence of HPV (*n* = 29)	1—no	1		
	2—yes	0.736	0.153–3.546	0.703
Past or active smoking	1—no	1		
	2—yes	2.156	0.264–17.624	0.474
Immunosuppressive therapy	0—NR	1		
	1—none	0.000	0.000	0.984
	2—non biologics	2.262	0.802–6,385	0.123
	3—biologics	3.428	1.031–11.404	0.045
	4—combined biologic and nonbiologic	13.970	1.706–114.368	0.014
Morphology (*n* = 53)	1—ulcer	10.378	1.386–77.727	0.023
	2—nodule/plaque	2.742	0.246–30.567	0.412
	3—verrucous	1		
Histologic grade (Broder) (*n* = 68)	1—1 (well)	1		
	2—2 (mod)	1.553	0.586–4.114	0.376
	3—3 (poorly)	7.186	2.835–18.219	0.000
	4—1(V) (well-verrucous)	0.346	0.071–1.684	0.189
	5—in situ	0.000	0.000	0.982
Palpable lymphadenopathy *(n* = 20)	1—no	1		
	2—yes	41.694	0.168–10335.3	0.185
Presence of nodal metastasis (*n* = 71)	1—no	1		
	2—yes	9.669	3.884–24.07	0.000
Site of nodal metastasis (*n* = 22)	1—inguinal	1		
	2—other	-		
	3—multiple (>1)	0.911	0.261–3.175	0.883
Presence of distant metastasis (*n* = 72)	1—no	1		
	2—yes	4.261	2.069–8.775	0.000
Site of distant metastasis (*n* = 20)	1—lung	1		
	2—bone	0.351	0.039–3.190	0.352
	3—other	2.390	0.545–10.47	0.248
	4—multiple (>1)	0.931	0.289–2.99	0.905
Excision (*n* = 91)	2—yes	1		
	1—no	8.184	4.108–16.30	0.000
Radiotherapy (*n* = 37)	1—no	1		
	2—yes	2.469	0.703–8.671	0.159
Chemotherapy (*n* = 32)	1—no	1		
	2—yes	2.082	0.752–5.764	0.158
Recurrent cSCC (*n* = 32)	1—no	1		
	2—yes, same site	5.218	1.91–14.28	0.001
	3—yes, different site	7.944	1.53–41.34	0.014

Legend. OR: odds ratio; CI: confidence interval; n: number of cases included (no missing data); cSCC: cutaneous squamous cell carcinoma; HS: hidradenitis suppurativa; HPV: human papillomavirus; NR: not reported.

## Data Availability

The data presented in this study are available in this article (and supplementary material).

## Supplementary Materials

The following are available online at https://www.mdpi.com/2072-6694/13/5/1153/s1, Table S1: Methodological quality assessment.

## References

[1] Saunte D.M.L., Jemec G.B.E. (2017). Hidradenitis Suppurativa: Advances in Diagnosis and Treatment. JAMA.

[2] Chapman S., Delgadillo D., Barber C., Khachemoune A. (2018). Cutaneous Squamous Cell Carcinoma Complicating Hidradenitis Suppurativa: A Review of the Prevalence, Pathogenesis, and Treatment of This Dreaded Complication. Acta Dermatovenerol. Alp. Pannonica Adriat..

[3] Lee E.Y., Alhusayen R., Lansang P., Shear N., Yeung J. (2017). What Is Hidradenitis Suppurativa?. Can. Fam. Phys..

[4] Li X., Jiang L., Huang Y., Ren Z., Liang X., Wang P. (2020). A Gene Dysfunction Module Reveals the Underlying Pathogenesis of Hidradenitis Suppurativa: An Update. Austr. J. Dermatol..

[5] Jfri A.H., O’Brien E.A., Litvinov I.V., Alavi A., Netchiporouk E. (2019). Hidradenitis Suppurativa: Comprehensive Review of Predisposing Genetic Mutations and Changes. J. Cutan. Med. Surg..

[6] Huang C., Lai Z., He M., Zhai B., Zhou L., Long X. (2017). Successful Surgical Treatment for Squamous Cell Carcinoma Arising from Hidradenitis Suppurativa: A Case Report and Literature Review. Medicine (Baltimore).

[7] Lavogiez C., Delaporte E., Darras-Vercambre S., Martin De Lassalle E., Castillo C., Mirabel X., Laurent F., Patenotre P., Gheit T., Talmant J.C. (2010). Clinicopathological Study of 13 Cases of Squamous Cell Carcinoma Complicating Hidradenitis Suppurativa. Dermatology (Basel).

[8] Hendricks A.J., Hsiao J.L., Lowes M.A., Shi V.Y. (2019). A Comparison of International Management Guidelines for Hidradenitis Suppurativa. DRM.

[9] Barresi V., Vitarelli E., Barresi G. (2008). Acne Inversa Complicated by Squamous Cell Carcinoma in Association with Diffuse Malignant Peritoneal Mesothelioma Arising in the Absence of Predisposing Factors: A Case Report. J. Cutan. Pathol..

[10] Ben A.J., Bouasker I., Najah H., Zribi H., Bedoui R., Guesmi F., Hani M.A., Nouira R., Zoghlami A., Najah N. (2008). Squamous Cell Carcinoma Arising in Verneuil’s Disease. Tunis Med..

[11] Ben Said B., Maitre S., Perrot J.-L., Labeille B., Cambazard F. (2010). Syndrome Paranéoplasique Hypercalcémie–Hyperleucocytose Au Cours Des Carcinomes Épidermoïdes Cutanés. À Propos de Deux Observations. La Revue de Méd. Interne.

[12] Brown M.D., Zachary C.B., Grekin R.C., Swanson N.A. (1988). Genital Tumors: Their Management by Micrographic Surgery. J. Am. Acad. Dermatol..

[13] Chicarilli Z.N.M.D. (1987). Follicular Occlusion Triad: Hidradenitis Suppurativa, Acne Conglobata, and Dissecting Cellulitis of the Scalp. Ann. Plast. Surg..

[14] Dessinioti C., Plaka M., Zisimou C., Christofidou E., Antoniou C., Stratigos A.J. (2017). Advanced Squamous Cell Carcinoma of the Axillae Mimicking Hidradenitis Suppurativa. J. Eur. Acad. Dermatol. Venereol..

[15] Giesey R., Delost G.R., Honaker J., Korman N.J. (2017). Metastatic Squamous Cell Carcinoma in a Patient Treated with Adalimumab for Hidradenitis Suppurativa. JAAD Case Rep..

[16] Gur E., Neligan P.C., Shafir R., Reznick R., Cohen M., Shpitzer T. (1997). Squamous Cell Carcinoma in Perineal Inflammatory Disease. Ann. Plast. Surg..

[17] Harview C.L., Truong A.K., Worswick S.D., Sarantopoulos G.P., Hsiao J.L. (2018). Squamous Cell Carcinoma of the Perineum Masquerading as Necrotizing Hidradenitis Suppurativa. Dermatol. Online J..

[18] Jourabchi N., Fischer A.H., Cimino-Mathews A., Waters K.M., Okoye G.A. (2017). Squamous Cell Carcinoma Complicating a Chronic Lesion of Hidradenitis Suppurativa: A Case Report and Review of the Literature. Int. Wound J..

[19] Juviler P.G., Patel A.P., Qi Y. (2019). Infiltrative Squamous Cell Carcinoma in Hidradenitis Suppurativa: A Case Report for Early Surgical Intervention. Int. J. Surg. Case Rep..

[20] Kohorst J.J., Shah K.K., Hallemeier C.L., Baum C.L., Davis M.D.P. (2019). Squamous Cell Carcinoma in Perineal, Perianal, and Gluteal Hidradenitis Suppurativa: Experience in 12 Patients. Dermatol. Surg..

[21] Maalouf E., Faye O., Poli F., Cosnes A., Revuz J. (2006). Carcinome Épidermoïde Mortel Sur Hidradénite Suppurée Après Traitement Par Infliximab. Ann. Dermatol. Vénéréol..

[22] Makris G.-M., Poulakaki N., Papanota A.-M., Kotsifa E., Sergentanis T.N., Psaltopoulou T. (2017). Vulvar, Perianal and Perineal Cancer After Hidradenitis Suppurativa: A Systematic Review and Pooled Analysis. Dermatol. Surg..

[23] Manolitsas T., Biankin S., Jaworski R., Wain G. (1999). Vulval Squamous Cell Carcinoma Arising in Chronic Hidradenitis Suppurativa. Gynecol. Oncol..

[24] McArdle D.J.T., McArdle J.P., Lee F., Mignanelli E.D. (2017). Rare “Inverted” Verrucous Carcinoma (Carcinoma Cuniculatum) of the Sacrogluteal Region: Case Report and Literature Review. Int. J. Surg. Pathol..

[25] Miura T., Ishikawa M., Mori T., Hanami Y., Ohtsuka M., Yamamoto T. (2018). Huge Squamous Cell Carcinoma Arising on Severe Hidradenitis Suppurativa. Actas Dermosifiliogr.

[26] Montagliani L., Monneuse O., Braye F., Barth X., Claudy A., Tissot E. (2005). Maladie de Verneuil et Cancer. J. De Chirurgie J. CHIR.

[27] Obredor C., Palermo M., Zorraquín C., Albertengo J.C. (2009). Perineal suppurative hidradenitis and carcinoma. A case report. Acta Gastroenterol. Latinoam..

[28] Pena Z.G., Sivamani R.K., Konia T.H., Eisen D.B. (2015). Squamous Cell Carcinoma in the Setting of Chronic Hidradenitis Suppurativa; Report of a Patient and Update of the Literature. Dermatol. Online J..

[29] Pérez-Diaz D., Calvo-Serrano M., Mártinez-Hijosa E., Fuenmayor-Valera L., Muñoz-Jiménez F., Turégano-Fuentes F., Del Valle E. (1995). Squamous Cell Carcinoma Complicating Perianal Hidradenitis Suppurativa. Int. J. Colorectal Dis..

[30] Pitch M.A., Bryan D.J., McMillan J., Chavez L., Hammes S.R., Scott G., Mercurio M.G., Somers K.E. (2018). A Fatal Case of Parathyroid Hormone-Related Peptide (PTHrP)-Producing Squamous Cell Carcinoma Arising in the Context of Long-Standing Hidradenitis Suppurativa. JAAD Case Rep..

[31] Powell H.B., Googe P.B., Sayed C.J. (2018). Squamous Cell Carcinoma Arising in a Chronic Perineal Wound in a Patient with Long-Standing Cutaneous Crohn’s Disease. JAAD Case Rep..

[32] Rekawek P., Mehta S., Andikyan V., Harmaty M., Zakashansky K. (2016). Squamous Cell Carcinoma of the Vulva Arising in the Setting of Chronic Hidradenitis Suppurativa: A Case Report. Gynecol. Oncol. Rep..

[33] Rosen T. (1986). Squamous Cell Carcinoma: Complication of Chronic Skin Disorders in Black Patients. J. Natl. Med. Assoc..

[34] Roy C.F., Roy S.F., Ghazawi F.M., Patocskai E., Bélisle A., Dépeault A. (2019). Cutaneous Squamous Cell Carcinoma Arising in Hidradenitis Suppurativa: A Case Report. SAGE Open Med. Case Rep..

[35] Segura Palacios J.M., García Montero P., Fúnez Liébana R., Repiso Jiménez J.B. (2018). Human Papilloma Virus and the Risk of Squamous Cell Carcinoma Arising in Hidradenitis Suppurativa. Actas Dermo-Sifiliográficas (English Edition).

[36] Sevray M., Dupré P.-F., Le Flahec G., Trimaille A., Misery L., Brenaut E. (2019). Vulvar Squamous Cell Carcinoma Complicating Hidradenitis Suppurativa in a Young Woman. JAAD Case Rep..

[37] Talmant J.-C., Bruant-Rodier C., Nunziata A.C., Rodier J.F., Wilk A. (2006). Squamous cell carcinoma arising in Verneuil’s disease: Two cases and literature review. Ann. Chir. Plast. Esthet..

[38] Yatim A., Bohelay G., Grootenboer-Mignot S., Prost-Squarcioni C., Alexandre M., Le Roux-Villet C., Martin A., Maubec E., Caux F. (2019). Paraneoplastic Pemphigus Revealed by Anti-Programmed Death-1 Pembrolizumab Therapy for Cutaneous Squamous Cell Carcinoma Complicating Hidradenitis Suppurativa. Front. Med..

[39] Yen C.-F., Chang Y.-Y., Lee Y.-Y. (2018). Image Gallery: Squamous Cell Carcinoma Arising in Long-Standing Hidradenitis Suppurativa. Br. J. Dermatol..

[40] Zhang L.-Q., Tan C. (2017). Squamous Cell Carcinoma Arising in Chronic Hidradenitis Suppurativa: A Lethal Complication to Be Avoided. Acta Oncol..

[41] Murad M.H., Sultan S., Haffar S., Bazerbachi F. (2018). Methodological Quality and Synthesis of Case Series and Case Reports. BMJ Evid. Based Med..

[42] Short K.A., Kalu G., Mortimer P.S., Higgins E.M. (2005). Vulval Squamous Cell Carcinoma Arising in Chronic Hidradenitis Suppurativa. Clin. Exp. Dermatol..

[43] Grewal N., Wan D., Roostaeian J., Rubayi S. (2010). Marjolin Ulcer in Hidradenitis Suppurativa: Case Reports. Ann. Plast. Surg..

[44] Rogers H.W., Weinstock M.A., Feldman S.R., Coldiron B.M. (2015). Incidence Estimate of Nonmelanoma Skin Cancer (Keratinocyte Carcinomas) in the U.S. Population, 2012. JAMA Dermatol..

[45] Novick M., Gard D.A., Hardy S.B., Spira M. (1977). Burn Scar Carcinoma: A Review and Analysis of 46 Cases. J. Trauma.

[46] Knackstedt T.J., Collins L.K., Li Z., Yan S., Samie F.H. (2015). Squamous Cell Carcinoma Arising in Hypertrophic Lichen Planus: A Review and Analysis of 38 Cases. Dermatol. Surg..

[47] Boozalis E., Shah A.A., Wigley F., Kang S., Kwatra S.G. (2019). Morphea and Systemic Sclerosis Are Associated with an Increased Risk for Melanoma and Nonmelanoma Skin Cancer. J. Am. Acad. Dermatol..

[48] Xiang F., Lucas R., Hales S., Neale R. (2014). Incidence of Nonmelanoma Skin Cancer in Relation to Ambient UV Radiation in White Populations, 1978-2012: Empirical Relationships. JAMA Dermatol..

[49] Wikonkal N.M., Brash D.E. (1999). Ultraviolet Radiation Induced Signature Mutations in Photocarcinogenesis. J. Investig. Dermatol. Symp. Proc..

[50] Fabbrocini G., Ruocco E., De Vita V., Monfrecola G. (2017). Squamous Cell Carcinoma Arising in Long-Standing Hidradenitis Suppurativa: An Overlooked Facet of the Immunocompromised District. Clin. Dermatol..

[51] Chu E.Y., Kovarik C.L., Lee R.A. (2013). Lymphedematous Verrucous Changes Simulating Squamous Cell Carcinoma in Long-Standing Hidradenitis Suppurativa. Int. J. Dermatol..

[52] Foster D.S., Jones R.E., Ransom R.C., Longaker M.T., Norton J.A. (2018). The Evolving Relationship of Wound Healing and Tumor Stroma. JCI Insight.

[53] Rønnov-Jessen L., Petersen O.W. (1993). Induction of Alpha-Smooth Muscle Actin by Transforming Growth Factor-Beta 1 in Quiescent Human Breast Gland Fibroblasts. Implications for Myofibroblast Generation in Breast Neoplasia. Lab. Investig..

[54] Pierce G.F., Mustoe T.A., Altrock B.W., Deuel T.F., Thomason A. (1991). Role of Platelet-Derived Growth Factor in Wound Healing. J. Cell. Biochem..

[55] zur Hausen H. (2009). Papillomaviruses in the Causation of Human Cancers—A Brief Historical Account. Virology.

[56] Miller D.L., Puricelli M.D., Stack M.S. (2012). Virology and Molecular Pathogenesis of HPV (Human Papillomavirus)Associated Oropharyngeal Squamous Cell Carcinoma. Biochem. J..

[57] Flores R., Lu B., Beibei L., Nielson C., Abrahamsen M., Wolf K., Lee J.-H., Harris R.B., Giuliano A.R. (2008). Correlates of Human Papillomavirus Viral Load with Infection Site in Asymptomatic Men. Cancer Epidemiol. Biomark. Prev..

[58] Pham C.T., Juhasz M., Sung C.T., Mesinkovska N.A. (2020). The Human Papillomavirus Vaccine as a Treatment for Human Papillomavirus–Related Dysplastic and Neoplastic Conditions: A Literature Review. J. Am. Acad. Dermatol..

[59] Mejilla A., Li E., Sadowski C.A. (2017). Human Papilloma Virus (HPV) Vaccination: Questions and Answers. Can. Pharm. J..

[60] Panelos J., Massi D. (2009). Emerging Role of Notch Signaling in Epidermal Differentiation and Skin Cancer. Cancer Biol. Ther..

[61] Montaudié H., Chiaverini C., Sbidian E., Charlesworth A., Lacour J.-P. (2016). Inherited Epidermolysis Bullosa and Squamous Cell Carcinoma: A Systematic Review of 117 Cases. Orphanet. J. Rare Dis..

[62] Combemale P., Bousquet M., Kanitakis J., Bernard P. (2007). Malignant Transformation of Leg Ulcers: A Retrospective Study of 85 Cases. J. Eur. Acad. Dermatol. Venereol..

[63] Schmults C.D., Alam M., Chen P.-L., Daniels G.A., DiMaio D., Farma J.M., Ghosh K., Harms K., Sekulic A., Stebbins W. (2019). NCCN Guidelines Index Table of Contents. https://www.nccn.org/professionals/physician_gls/default.aspx.

[64] Migden M.R., Rischin D., Schmults C.D., Guminski A., Hauschild A., Lewis K.D., Chung C.H., Hernandez-Aya L., Lim A.M., Chang A.L.S. (2018). PD-1 Blockade with Cemiplimab in Advanced Cutaneous Squamous-Cell Carcinoma. N. Engl. J. Med..

[65] Maubec E., Petrow P., Scheer-Senyarich I., Duvillard P., Lacroix L., Gelly J., Certain A., Duval X., Crickx B., Buffard V. (2011). Phase II Study of Cetuximab as First-Line Single-Drug Therapy in Patients with Unresectable Squamous Cell Carcinoma of the Skin. J. Clin. Oncol..

