# Supplementary material for: Cutaneous Squamous Cell Carcinoma in Patients with Hidradenitis Suppurativa
Source: Cancers (Basel). 2021 Mar 8;13(5):1153. doi: 10.3390/cancers13051153 (PMC7962537; doi:10.3390/cancers13051153)
Supplement: Supplementary file 1 [file cancers-13-01153-s001.pdf]

Table S1. Methodological quality assessment.

Study	Year	Design	Q1	Q2	Q3	~~Q4~~	~~Q5~~	~~Q6~~	Q7	Q8
**Anderson**	1958	Case series	N	Y	Y	~~N~~	~~X~~	~~X~~	Y	Y
**Jackman**	1959	Case series	N	Y	Y	~~N~~	~~X~~	~~X~~	Y	N
**Donsky**	1964	Case report	N	Y	Y	~~N~~	~~X~~	~~X~~	Y	Y
**Humphrey**	1969	Case report	N	Y	Y	~~N~~	~~X~~	~~X~~	Y	Y
**Gordon**	1977	Case report	N	Y	Y	~~N~~	~~X~~	~~X~~	Y	Y
**Thornton**	1978	Case report	Y	Y	Y	~~N~~	~~X~~	~~X~~	Y	N
**Alexander**	1979	Case report	N	Y	Y	~~N~~	~~X~~	~~X~~	Y	Y
**Johnston**	1980	Case series	N	Y	Y	~~N~~	~~X~~	~~X~~	Y	Y
**Mora**	1981	Case series	Y	Y	Y	~~Y~~	~~X~~	~~X~~	Y	N
**Black**	1982	Case report	N	Y	Y	~~N~~	~~X~~	~~X~~	Y	Y
**Sparks**	1985	Case report	N	Y	Y	~~N~~	~~X~~	~~X~~	Y	Y
**Crevé**	1986	Case series	N	Y	Y	~~N~~	~~X~~	~~X~~	Y	N
**Rosen**	1986	Case report	N	Y	Y	~~N~~	~~X~~	~~X~~	Y	Y
**Zachary**	1987	Case report	N	Y	Y	~~N~~	~~X~~	~~X~~	Y	Y
**Chicarilli**	1987	Case report	N	Y	Y	~~N~~	~~X~~	~~X~~	Y	Y
**Brown**	1988	Case report	Y	Y	Y	~~N~~	~~X~~	~~X~~	Y	N
**Martinez**	1989	Case report	N	Y	Y	~~N~~	~~X~~	~~X~~	Y	Y
**Anstey**	1989	Case report	N	Y	Y	~~N~~	~~X~~	~~X~~	Y	Y
**Weber**	1990	Case report	N	Y	Y	~~N~~	~~X~~	~~X~~	Y	Y
**Mendonca**	1991	Case report	N	Y	Y	~~N~~	~~X~~	~~X~~	Y	Y
**Williams**	1991	Case report	N	Y	Y	~~N~~	~~X~~	~~X~~	Y	N
**Welsh**	1993	Case report	N	Y	Y	~~N~~	~~X~~	~~X~~	Y	Y
**Shukla**	1995	Case report	N	Y	Y	~~N~~	~~X~~	~~X~~	Y	Y
**Perez-Diaz**	1995	Case report	N	Y	Y	~~N~~	~~X~~	~~X~~	Y	Y
**Dufresne**	1996	Case report	N	Y	Y	~~N~~	~~X~~	~~X~~	Y	Y
**Malaguarmera**	1996	Case report	N	Y	Y	~~N~~	~~X~~	~~X~~	Y	Y
**Li**	1997	Case report	N	Y	Y	~~N~~	~~X~~	~~X~~	Y	Y
**Gur**	1997	Case series	N	Y	Y	~~N~~	~~X~~	~~X~~	Y	Y
**Ritz**	1998	Case report	N	Y	Y	~~N~~	~~X~~	~~X~~	Y	Y
**Nijhawan**	1998	Case report	N	Y	Y	~~N~~	~~X~~	~~X~~	Y	Y
**Lin**	1999	Case report	N	Y	Y	~~N~~	~~X~~	~~X~~	Y	Y
**Manolitsas**	1999	Case report	N	Y	Y	~~N~~	~~X~~	~~X~~	Y	Y
**Cosman**	2000	Case report	N	Y	Y	~~N~~	~~X~~	~~X~~	Y	Y
**Ishizawa**	2000	Case report	N	Y	Y	~~N~~	~~X~~	~~X~~	Y	Y
**Lapins**	2001	Cohort study	Y	Y	Y	~~N~~	~~X~~	~~X~~	Y	Y
**Altunay**	2002	Case report	N	Y	Y	~~N~~	~~X~~	~~X~~	Y	Y
**Bocchini**	2003	Case report	N	Y	Y	~~N~~	~~X~~	~~X~~	Y	N
**Crain**	2005	Case series	N	Y	Y	~~N~~	~~X~~	~~X~~	Y	Y
**Rosenzweig**	2005	Case report	N	Y	Y	~~N~~	~~X~~	~~X~~	Y	Y
**Short**	2005	Case report	N	Y	Y	~~N~~	~~X~~	~~X~~	Y	Y
**Montagliani**	2005	Case report	N	Y	Y	~~N~~	~~X~~	~~X~~	Y	N
**Talmant**	2006	Case series	N	Y	Y	~~N~~	~~X~~	~~X~~	Y	Y
**Maalouf**	2006	Case report	N	Y	Y	~~N~~	~~X~~	~~X~~	Y	Y
**Maclean**	2007	Case series	N	Y	Y	~~N~~	~~X~~	~~X~~	Y	Y
**Kurokawa**	2007	Case series	N	Y	Y	~~N~~	~~X~~	~~X~~	Y	Y
**Barresi**	2007	Case report	N	Y	Y	~~N~~	~~X~~	~~X~~	Y	Y
**Constantinou**	2008	Case series	N	Y	Y	~~N~~	~~X~~	~~X~~	Y	Y
**Ben Achour**	2008	Case report	N	Y	Y	~~N~~	~~X~~	~~X~~	Y	Y
**Balik**	2009	Case report	Y	Y	Y	~~N~~	~~X~~	~~X~~	Y	N
**Chandramohan**	2009	Case report	N	Y	Y	~~N~~	~~X~~	~~X~~	Y	Y
**Ito**	2009	Case report	N	Y	Y	~~N~~	~~X~~	~~X~~	Y	Y
**Katz**	2009	Case report	N	Y	Y	~~N~~	~~X~~	~~X~~	Y	Y
**Miquel**	2009	Case report	N	Y	Y	~~N~~	~~X~~	~~X~~	Y	Y
**Obredor**	2009	Case report	N	Y	Y	~~N~~	~~X~~	~~X~~	Y	Y
**Lavogiez**	2010	Case series	Y	Y	Y	~~N~~	~~X~~	~~X~~	Y	Y

**Grewal**	2010	Case series	N	Y	Y	~~N~~	~~X~~	~~X~~	Y	Y
**Vogelaar**	2010	Case report	N	Y	Y	~~N~~	~~X~~	~~X~~	Y	Y
**Said**	2010	Case report	N	Y	Y	~~N~~	~~X~~	~~X~~	Y	N
**Büyükasik**	2011	Case report	Y	Y	Y	~~N~~	~~X~~	~~X~~	Y	N
**Losanoff**	2011	Case series	N	Y	Y	~~N~~	~~X~~	~~X~~	Y	Y
**Pagliarello**	2011	Case report	N	Y	Y	~~N~~	~~X~~	~~X~~	Y	Y
**Belli**	2012	Case series	N	Y	Y	~~N~~	~~X~~	~~X~~	Y	Y
**Herschel**	2014	Case report	N	Y	Y	~~N~~	~~X~~	~~X~~	Y	Y
**Matoso**	2014	Case report	Y	Y	Y	~~N~~	~~X~~	~~X~~	Y	N
**Chang**	2014	Case report	N	Y	Y	~~N~~	~~X~~	~~X~~	Y	Y
**Poh**	2014	Case report	N	Y	Y	~~N~~	~~X~~	~~X~~	Y	Y
**Scheinfeld**	2014	Case report	N	Y	Y	~~N~~	~~X~~	~~X~~	Y	Y
**Peña**	2015	Case report	N	Y	Y	~~N~~	~~X~~	~~X~~	Y	Y
**Joglekar**	2016	Case report	N	Y	Y	~~N~~	~~X~~	~~X~~	Y	Y
**Hessam**	2016	Case report	N	Y	Y	~~N~~	~~X~~	~~X~~	Y	Y
**Verdelli**	2016	Case report	N	Y	Y	~~N~~	~~X~~	~~X~~	Y	Y
**Jourabchi**	2016	Case report	N	Y	Y	~~N~~	~~X~~	~~X~~	Y	Y
**Rekawek**	2016	Case report	N	Y	Y	~~N~~	~~X~~	~~X~~	Y	Y
**Powell**	2017	Case report	N	Y	Y	~~N~~	~~X~~	~~X~~	Y	Y
**Yon**	2017	Case series	Y	Y	Y	~~N~~	~~X~~	~~X~~	Y	Y
**Giesey**	2017	Case report	N	Y	Y	~~N~~	~~X~~	~~X~~	Y	Y
**McArdle**	2017	Case report	N	Y	Y	~~N~~	~~X~~	~~X~~	Y	Y
**Zhang**	2017	Case report	N	Y	Y	~~N~~	~~X~~	~~X~~	Y	Y
**Huang**	2017	Case report	N	Y	Y	~~N~~	~~X~~	~~X~~	Y	Y
**Makris**	2017	Case report	Y	Y	Y	~~N~~	~~X~~	~~X~~	Y	Y
**Dessinioti**	2017	Case report	N	Y	Y	~~N~~	~~X~~	~~X~~	Y	Y
**Yen**	2018	Case report	N	Y	Y	~~N~~	~~X~~	~~X~~	Y	Y
**Miura**	2018	Case report	N	Y	Y	~~N~~	~~X~~	~~X~~	Y	Y
**Pitch**	2018	Case report	N	Y	Y	~~N~~	~~X~~	~~X~~	Y	Y
**Harview**	2018	Case report	N	Y	Y	~~N~~	~~X~~	~~X~~	Y	Y
**Segura Palacios**	2018	Case report	N	Y	Y	~~N~~	~~X~~	~~X~~	Y	Y
**Roy**	2019	Case report	N	Y	Y	~~N~~	~~X~~	~~X~~	Y	Y
**Yatim**	2019	Case report	N	Y	Y	~~N~~	~~X~~	~~X~~	Y	Y
**Sevray**	2019	Case report	N	Y	Y	~~Y~~	~~X~~	~~X~~	Y	Y
**Juviler**	2019	Case report	N	Y	Y	~~N~~	~~X~~	~~X~~	Y	Y
**Kohorst**	2019	Case series	Y	Y	Y	~~N~~	~~X~~	~~X~~	Y	Y

Legend: Y: yes, N: no, X: not assessed. **Q1.** Does the patient(s) represent(s) the whole experience of the investigator (center) or is the selection method unclear to the extent that other patients with similar presentation may not have been reported? **Q2.** Was the exposure adequately ascertained? **Q3.** Was the outcome adequately ascertained? **Q4.** Were other alternative causes that may explain the observation ruled out? **Q5.** Was there a challenge/rechallenge phenomenon? **Q6.** Was there a dose–response effect? **Q7.** Was follow-up long enough for outcomes to occur? **Q8.** Is the case(s) described with sufficient details to allow other investigators to replicate the research or to allow practitioners make inferences related to their own practice? We removed Q4, 5 and 6 because they were either not assessed or irrelevant to the included case reports and series; moreover, these questions are mostly applicable to studies reporting adverse drug events [9]. Reasons are as follows: Q4: Most cSCC are caused by chronic sun exposure; all cases of cSCC developing prior to HS or in an area unaffected by HS were excluded from this analysis. While it was not explicitly mentioned in many case reports or case series that other causes of cSCC were excluded, it is very plausible that cSCC developing in areas of long-standing HS could be secondary to chronic inflammation and aberrant wound healing pathways. Q5: A challenge/rechallenge phenomenon would not be possible to observe with the given exposure and outcome. Q6: A dose/response effect would not be possible with the given exposure and outcome.
